# Vegetable Oils and Their Use for Frying: A Review of Their Compositional Differences and Degradation

**DOI:** 10.3390/foods13244186

**Published:** 2024-12-23

**Authors:** Susana Abrante-Pascual, Barbara Nieva-Echevarría, Encarnacion Goicoechea-Oses

**Affiliations:** Department of Food Technology, Faculty of Pharmacy, Lascaray Research Center, University of the Basque Country (UPV/EHU), 01006 Vitoria-Gasteiz, Spain

**Keywords:** vegetable oils, frying, thermoxidation, polymerization, hydrolysis, *trans* fatty acids, cyclic fatty acids, monounsaturated oils, polyunsaturated oils, saturated oils

## Abstract

This review provides an overview of the main vegetable oils of different botanical origin and composition that can be used for frying worldwide (olive and extra-virgin olive oil, high-oleic sunflower oil, rapeseed oil, peanut oil, rice bran oil, sunflower oil, corn oil, soybean oil, cottonseed oil, palm oil, palm kernel oil and coconut oil) and their degradation during this process. It is well known that during this culinary technique, oil’s major and minor components degrade throughout different reactions, mainly thermoxidation, polymerization and, to a lesser extent, hydrolysis. If severe high temperatures are employed, isomerization to *trans* fatty acyl chains and cyclization are also possible. The factors conditioning frying medium degradation are addressed, including oil composition (unsaturation degree, fatty acyl chain length and “free” fatty acid content, and presence of beneficial and detrimental minor components), together with frying conditions and food characteristics. Likewise, this review also tackles how the frying oil and other processing conditions may impact on fried food quality (oil absorption, texture, flavor and color). Finally, potential health implications of fried food consumption are briefly reviewed.

## 1. Introduction

Frying is a fast and easy cooking method commonly used worldwide, in which the oil that is used as the heat transfer medium reaches high temperatures (150–190 °C). It provokes highly appreciated sensory properties in fried food, like golden color, crispy surface texture, juicy interior and characteristic flavor and aroma, which result in highly palatable foods [1,2]. It must be noted that frying is a very complex process in which heat and mass transfer phenomena occur between the food and the frying oil, and simultaneously the food dehydrates, absorbs oil and releases some of its lipids to the frying medium [3]. This oil absorption usually implies that fried food will have a higher caloric content, compared to the same food if cooked using another method in which oil is not employed as the heat transfer medium, such as steaming, boiling or roasting. Due to this, from a nutritional point of view, the consumption of fried foods is recommended on an occasional rather than a regular basis. Additionally, if reheated oils containing potentially toxic degradation compounds are used for deep frying, since they can be absorbed by the food, they may compromise its safety, as well as cause deterioration in the food sensory properties and shelf life during storage, which can result in economic losses for the food industry [4]. Thus, it is of paramount importance to select good-quality frying oils with a high stability and nutritional profile and also to choose the optimum frying conditions in order to obtain safer fried food with desirable sensory properties, because these aspects will have a great impact not only on the health of consumers, but also on the fried food industry.

In this context, this review deals with the composition in the major and minor components of the vegetable oils most frequently used for this culinary technique in different regions, the chemical reactions occurring in the oil during frying, the main factors conditioning frying medium degradation and finally, the influence of frying oil on food quality and a brief overview of the health implications of their consumption. Special attention will be paid to the fact that depending on the composition of the selected frying oil and also on the frying conditions applied, different degradation products can be generated and thus be absorbed by fried foods. As mentioned above, this fact will directly affect food quality and safety and ultimately consumers’ health.

## 2. Composition of Vegetable Oils Used for Frying

A wide variety of oils and fats can be used for frying depending on regional availability, culinary traditions and economic factors, among others. In Europe, sunflower oil predominates in the East, olive oil in the Mediterranean area and rapeseed oil in the North, although soybean oil is also extensively employed. In Africa, palm oil predominates in the sub-Saharan regions, peanut oil in West Africa and soybean oil is also used in some parts. In Asia, soybean oil is widely used, while palm oil is preferentially employed in the southeast, rapeseed oil in Japan and sunflower oil in Russia. In Oceania, rapeseed oil is mainly used, as in Canada, whereas in the rest of the Americas, soybean oil is the most common, along with palm oil in some parts of South America [5].

The common feature of all the above-mentioned oils is that triglycerides (TG) constitute the major components. Regarding the nature and proportion of the other minor constituents, these are highly dependent on the botanical origin and technological process of oil obtention. Among minor components usually present, there are sterols (mainly *beta*-sitosterol), terpenic lipids like vitamin E isoforms (mainly tocopherols and tocotrienols), carotenoids and squalene, as well as phenolic compounds, chlorophylls, phospholipids, waxes and trace metals, all of which play a crucial role in oil stability [6]. Vegetable oils used for frying usually undergo refining processes that result in the reduction of many of these minor compounds, some of them of nutritional interest and others showing detrimental effects on oil susceptibility to degradation [7,8,9]. Oil refining is performed to remove those compounds responsible for color, odor and taste that make the crude oil unacceptable to the consumer or that may compromise oil quality, technological performance and safety [6]. It must be noted that in the Mediterranean area, virgin or extra-virgin olive oil (EVOO) is traditionally used for frying [10].

Regarding oil’s major components, TG account for approximately 95–98% of the total lipid components. The fatty acids esterified with the glycerol backbone of TG, also called fatty acyl chains (FA), can be classified into either saturated (SFA) or unsaturated (UFA). The latter are further divided into monounsaturated fatty acyl chains (MUFA), characterized by a single double bond in their structure, like oleic (C18:1ω9), and into polyunsaturated fatty acyl chains (PUFA), which contain two or more double bonds, like linoleic (C18:2ω6) and linolenic (C18:3ω3). It must be noted that PUFA are especially prone to oxidation and thus in frying oil, the linolenic level is recommended to be below 3% [3,7]. Although SFA offer greater oxidative stability, as they are related to cardiovascular diseases, oils rich in them are less desirable from the nutritional point of view. However, it must be noted that in the last few years, controversy in this context is increasing, as reflected in recent reviews [11,12]. Moreover, their usefulness can be limited from the technological point of view, because in temperate climates, they can solidify during storage, leading, for instance, to pipe blockage in industrial frying [7,13]. Thus, it is important to select an appropriate frying oil, considering not only sociocultural and economic factors, but also the nutritional and technological point of view. Therefore, oils rich in MUFA are highly recommended for frying due to their high oxidative stability and greater interest than SFA-rich oils from the nutritional point of view.

Table 1 shows a summary of the composition of vegetable oils commonly used for frying worldwide, in major and minor components [14,15]. The compositional data shown were obtained from the standards established by the Codex Alimentarius Commission of the Food and Agriculture Organization (FAO) of the United Nations. These standards reflect a global representation of the typical composition of oils, based on data from different regions, including potential variations due to factors such as oilseed varieties, climate conditions and regional agricultural practices. As can be observed in Table 1, in some oils, remarkable variations have been reported, especially regarding the main FA content (like in peanut and maize oils) and also minor components (such as rice bran and maize oils). These differences in oil composition across regions could influence frying oil stability and the formation of specific degradation products during heating.

Furthermore, these vegetable oils can be divided into three groups depending on their source [6]. Firstly, by-products where the crop has been grown for another purpose other than obtaining oil, like rice bran oil (grain), corn or maize oil (grain), soybean oil (protein-rich grain) and cottonseed oil (fabric). Secondly, tree crops that are generally slow to mature but then produce crops regularly for many years, like olive oil, palm oil, palm kernel oil or coconut oil. And thirdly, crops that have to be replanted each year to produce an annual harvest, such as sunflower oil, rapeseed oil and peanut oil. A brief description of their composition is provided below.

### 2.1. Vegetable Oils Rich in MUFA

**Olive** oil, extracted from the fruit of the olive tree *Olea europaea*, is a fundamental component of the Mediterranean diet, ranking as the fourth most commonly used oil in Europe [5,16]. The main producer is Spain, followed by Italy, Turkey and Greece. Olive oil is particularly rich in MUFA, especially oleic (55–83%), and it also contains lower amounts of SFA (8.0–26.2%), mainly palmitic, and of essential PUFA omega-6 linoleic (3.5–21.0%) and omega-3 linolenic (less than 1.5%). Continued consumption of olive oil has been associated with several cardiovascular health benefits, related not only to this FA profile, but also to the presence of minor compounds that are potent bioactives, specially in virgin olive oil, such as phenolic compounds (oleuropein, hydroxytyrosol, tyrosol, oleocanthal, oleacein…) and squalene, together with other minor compounds also present in other vegetable oils like sterols and tocopherols (mainly *alpha*-tocopherol), among others [15,17,18,19]. It must be noted that in the last few years, special attention was paid to the health benefits attributed to EVOO polyphenols [19,20]. Due to this, the consumption of olive oil is increasing in Northern Europe, the United States of America (USA), Canada and other countries [6].

Compared to other unsaturated vegetable oils, olive oil has a much higher oxidative stability due to its lipid profile (high content of MUFA and presence of antioxidant components). Thus, as it is particularly stable during storage and heat treatment at high temperatures, olive oil is considered a premium frying oil [6,7,21,22,23]. In addition, it has been evidenced that if EVOO or virgin olive oil are used for frying, fried foods are enriched in the above-mentioned health-promoting bioactive compounds as a consequence of oil absorption, leading to the increase in fried food nutritional value and shelf life [24,25]. While in Anglo-Saxon countries frequent consumption of fried food is associated with a higher risk of cardiovascular diseases and prostate cancer, as well as overall mortality [26,27,28], such associations have not been found in Mediterranean countries [26,29,30]. This discrepancy could be partly explained by the type of oil used for frying [31], olive oil being the most commonly employed in Mediterranean countries. More recently, it has been reported that the consumption of food fried in olive oil can be associated with certain health benefits, like a delayed unhealthy aging, which is linked to several adverse health outcomes in older adults like institutionalization, hospitalization or even death [32]. Unfortunately, olive oil availability worldwide is limited.

**High-oleic sunflower** oil is another oil rich in MUFA, obtained from a specific strain of seeds that sunflower breeders created to increase the oxidative stability of traditional sunflower oil, and thus improve its frying performance [33,34,35,36]. The main producing countries are France and Ukraine, its production being less than 10% of that of sunflower oil. As can be observed in Table 1, this oil contains very high proportions of oleic (75.0–90.7%), along with smaller quantities of linoleic (2.1–17.0%) and SFA (6.2–13.9%). The content of minor components, such as sterols and tocopherols (mainly *alpha*-tocopherol), is similar to that of traditional refined sunflower oil. Although high-oleic sunflower oil shows an FA profile similar to that of olive oil, it lacks the polyphenols that are present in EVOO, which contribute to its quality, stability and nutritional value.

**Rapeseed** oil is obtained from the seed of two plant species, *Brassica napus* and *Brassica rapa*, and is currently the third most abundant oilseed crop in the world (after palm and soya) [5]. Naturally, rapeseed produces an oil rich in erucic (C22:1ω9, ~50%), which can be potentially toxic if present in high amounts, and also in anti-nutritive compounds like glucosinolates. For this reason, since 1970, seed varieties with a reduced content in erucic (≤5%) and glucosinolates have been selected [37], some of them known as “canola” oil (acronym for “Canada Oil Low Acid”). Even so, the oils of these varieties are refined to lower their content even further. The main producing countries are Germany, France and the United Kingdom in Europe, together with Canada, China and India [5].

Regarding its composition, refined low-erucic acid rapeseed oil (canola) is rich in MUFA, oleic being the most abundant (51–70%). It also contains low proportions of SFA (3.5–12.3%) and much higher of PUFA, which are linoleic (15–30%) and linolenic (5–14%). This latter high content of linolenic can make this oil prone to oxidation and off-flavor development [7], similar to what happens with soybean oil (see Table 1) [38]. For an improved oxidative stability during deep frying, high-oleic and partially hydrogenated rapeseed oils were developed [39], but the use of the latter is not encouraged due to the potential presence of *trans* FA [33,36]. Among rapeseed oil’s minor components, tocopherols (mainly *gamma*-tocopherol) and sterols can be found, brassicasterol being noteworthy, present almost exclusively in this type of oil.

**Peanut** oil, also known as groundnut oil, is derived from *Arachis hypogaea,* which botanically belongs together with soybean to the *Leguminosae* family [40,41,42]. The most widely consumed form of this oil is refined. The main producing country is China, followed by India [5]. As can be observed in Table 1, this oil is rich in MUFA, mainly oleic (35–80%). It also contains considerable proportions of PUFA, particularly linoleic (4–43%), linolenic being almost absent (≤0.5%), which makes it a good alternative for frying [6,43]. It contains relatively low levels of SFA (8.7–27.7%), and regarding the minor components, it contains lower levels of sterols and tocopherols (mainly *gamma-* and *alpha*-tocopherol in almost equal amounts) than other oils [14,41]. In the last few years, special attention has been paid to the presence in the oil of allergenic proteins and mycotoxins derived from toxigenic fungi, particularly aflatoxins; both of them are eliminated during the oil refining process [44].

**Rice bran** oil is extracted from the germ and dark-colored bran of rice grain (*Oryza sativa* L.) and is popular as cooking oil in several Asian countries of the Pacific area, India, China and Japan being the main producers [5]. Before the extraction process using solvents, the inactivation of rice bran lipase is required due to oil’s high susceptibility to enzymatic degradation. Refined rice bran oil is characterized by similar proportions of MUFA, mainly oleic (38.0–48.0%), and PUFA, primarily linoleic (21.0—42.0%). It also contains lower levels of SFA, predominantly palmitic (14.0–23.0%), and smaller amounts of linolenic (0.1–2.9%). Concerning minor components, rice bran oil is one of the few oils containing appreciable concentrations of tocotrienols, along with palm oil, as well as high proportions of *alpha*-tocopherol. This oil stands out for its elevated sterol content (see Table 1), largely attributed to its abundance of *gamma*-oryzanol, a group of ferulic acid esters of triterpene alcohols and plant sterols that are known for exhibiting antioxidant activity [6,7].

### 2.2. Vegetable Oils Rich in PUFA

**Sunflower** oil, derived from the seeds of the sunflower *Helianthus annuus*, is the fourth most abundant oilseed crop in the world and one of the most widely consumed edible oils in Europe [5,45]. The main producing countries are Russia, Ukraine and Argentina. It is characterized by its high content of PUFA, mainly linoleic (45.4–74.0%), linolenic being practically absent (lower than 0.3%). It contains lower proportions of oleic (14–43%), and much lower of SFA (8.1–16.9%). Due to this high content of PUFA, sunflower oil shows a poor frying performance, and this is why the above-mentioned high-oleic sunflower oil was developed [7,33,34,35]. It should be noted that sunflower oil is a very important source of vitamin E, especially in the form of *alpha*-tocopherol. As for the presence of other minor components, it also contains sterols, among others.

**Corn** or **maize** is one of the three major cereal grains grown in the world, along with rice and wheat. Its oil is obtained from the germ, as a by-product of the processing of corn into maize starch, the main producers being the USA, China and Brazil [5]. Its FA composition is characterized by a high content of PUFA, particularly linoleic (34.0–65.6%), linolenic being in much lower proportions (<2%). The main MUFA present is oleic (20.0–42.2%), and SFA account for 8.9–22.4%. This FA profile of corn oil is very similar to that of sunflower oil (see Table 1). As for corn oil’s minor components, it is remarkable that in comparison with other vegetable oils, with the exception of rice bran oil, it is especially rich in sterols free and esterified with ferulic acid. Likewise, it contains large amounts of vitamin E (mainly *gamma*-tocopherol, although tocotrienols are also present) [6,9]. In some regions, like North America, the use of refined corn oil for frying has increased in the last few years, due to the restriction of partially hydrogenated fats for this purpose as they can contain *trans* FA [46,47].

**Soybean** oil is the second most abundant oilseed crop in the world. It is extracted from the seeds of the soybean plant (*Glycine max*) through crushing and refining processes, China, the USA and Brazil being the main producers [5]. As for its composition in major lipid components, it is rich in PUFA, especially in linoleic (48–59%), also containing relatively high proportions of linolenic (4.5–11.0%). Soybean oil also contains MUFA, mainly oleic (17–30%) and much lower proportions of SFA (10.1–21.0%). In line with that commented before on rapeseed oil (see Table 1), due to this high content in linolenic [7,38] for an improved oxidative stability during frying, high-oleic and partially hydrogenated soybean oils were developed [33,36]. Among its minor components, sterols and high proportions of vitamin E (mainly *gamma*-tocopherol and also tocotrienols) can be cited. According to the European Food Safety Authority (EFSA), during this refining process, the presence of allergenic proteins is also significantly reduced, and therefore fully refined soybean oil is unlikely to cause allergic reactions in most individuals with a soybean allergy [48].

**Cottonseed** oil is extracted from the seeds of cotton plants *Gossypium hirsutum* and *G. herbaceum* and usually refined. It is widely used for culinary purposes, mainly in Asia, the main producers being China and India [5,49]. The oil has a high content of PUFA, mainly linoleic (46.7–58.2%), along with moderate amounts of SFA, primarily palmitic (21.4–26.4%), and of MUFA, mainly oleic (14.7–21.7%). As for its minor components, it contains sterols and vitamin E, especially *gamma-* and *alpha*-tocopherol. One of the challenges regarding cottonseed oil is the presence of gossypol, a toxic phenolic compound, which must be kept within safe limits or eliminated. To mitigate this issue, genetic modification and other technological approaches have been developed to reduce gossypol levels in the oil, such as oil refining [6,42,45].

### 2.3. Vegetable Oils Rich in SFA

The oil palm, *Elaeis guineensis*, is a perennial plant native to West Africa. Two types of oil are produced from the fruit of the palm, depending on the part of the fruit used: palm oil, which is obtained from the mesocarp (the fibrous, orange-colored outer pulp of the fruit) and palm kernel oil, which is extracted from the white kernel found inside the shell of the palm fruit [6,7,50]. Indonesia and Malaysia are the main producers of these oils worldwide. These palm-derived oils are usually subjected to a refining process prior to commercialization, which can cause their bioactive minor components to be reduced [8].

As shown in Table 1, these oils have very different FA compositions. **Palm kernel** oil is very rich in SFA (71.5–97.6%), mainly medium-chain SFA lauric (C12:0, 45–55%), and also myristic (14–18%) and palmitic (6.5–10.0%). Among MUFA, oleic stands out (12–19%), while it contains only up to 3.7% PUFA, primarily linoleic and linolenic being almost absent (<0.2%). Due to this high content of lauric FA, palm kernel oil and coconut oil are also called “lauric oils” [6]. They contain lower proportions than other oils of sterols and much lower of tocopherols, *beta* and *gamma* isoforms being in similar proportions.

On the other hand, **palm** oil has a much more balanced FA composition, almost half of them being saturated and the other half unsaturated [51]. The SFA palmitic (39.3–47.5%) and the MUFA oleic (36–44%) are the most abundant, followed by linoleic (9–12%) and stearic (3.5–6.0%). As for its minor components, palm oil is rich in carotenoids such as *alpha-* and *beta*-carotene (precursors of vitamin A that give its characteristic red color to virgin palm oil). It also contains lower levels of sterols than other oils, but, regarding vitamin E, it is characterized by high levels of tocotrienols, like rice bran oil [50].

To meet diverse market demands, palm oil and palm kernel oil are often fractionated during the refining process into two main products: olein, which is the lower melting liquid fraction, and stearin, the higher melting solid fraction. These fractions have specific applications in food processing and industrial frying, with olein being particularly favored for frying due to its stability at high temperatures and liquid state at room temperature [7,50]. It must be noted that oils and fats derived from the oil palm plant are the most widely used worldwide. They are generally used in the food industry for the production of margarines, shortenings and as frying oils, being especially useful as an ingredient in the production of fat-rich foods such as cookies, ice cream or cream fillings.

In recent years, the use and consumption of these oils and fats derived from the palm plant has caused great controversy, mainly due to their high palmitic FA content and its potential relation to cardiovascular disease [51,52] and to the environmental damage (deforestation, loss of animal and plant biodiversity, etc.) caused by the excessive and unsustainable cultivation of these oil palm plants, mainly in Southeast Asia [53]. Furthermore, in relation to refined palm oil, special attention has been paid in recent years to the presence of the toxic compounds glycidol, 3-monochloropropane-1,2-diol (3-MCPD) and their esters, although the authorities have already implemented measures to reduce their content [54]. The International Agency for Research on Cancer (IARC), a World Health Organization (WHO) agency, has classified glycidol as “probably carcinogenic to humans” (Group 2A) and 3-MCPD as “possible human carcinogen” (Group 2B) [55]. It should be remembered that these compounds can be generated during the refining process of any vegetable oil at excessive temperatures, and not only during that of palm oil.

**Coconut** oil, sometimes called coconut fat, is obtained from the kernel of the nut of the coconut palm (*Cocos nucifera* L.). In the last decade, its production and consumption has increased significantly, with the Philippines and Indonesia being the main producers worldwide [5,56]. With regard to the composition in major components, it contains around 90% of SFA, which is significantly greater than in other commonly consumed vegetable oils, even more than palm kernel oil [57]. Among them, the high percentage of medium-chain SFA, which account for approximately 70% of the total SFA, is noteworthy: lauric (45.1–53.2%), caprylic (C8:0, 4.6–10.0%) and capric (C10:0, 5–8%). As mentioned above, it is because of this high-lauric FA content that coconut oil and palm kernel oil are commonly referred to as “lauric oils” [6]. As these medium-chain FA have a lower smoke point than long-chain FA, these oils are not recommended for frying [58]. Oil’s smoke point will be addressed below in Section 4.1.2. Despite this limitation, coconut oil is still used as a frying medium in certain regions at domestic level, mainly in Southeast Asia, where there is a cultural preference for it [6].

It should be noted that in coconut oil, the presence of UFA is very small, oleic being the main one (5–10%), followed by small proportions of the essential FA linoleic (1.0–2.5%). Despite being exceptionally rich in SFA, the lipid profile of coconut oil is different from that of animal fats, since the latter are rich in long-chain SFA, mainly palmitic (C16:0) and stearic (C18:0), whose metabolism differs from that of medium-chain SFA: the SFA lauric (C12:0) is rapidly absorbed and transported directly to the liver, where it is oxidized for energy production and it is not used as a substrate for fat accumulation [57]. Regarding minor components, coconut oil contains sterols, and significantly lower concentrations of vitamin E than in other vegetable oils (<50 mg/kg crude oil), *alpha*-tocotrienol being the main isoform.

It is therefore evident that all over the world, oils of very different composition are used for frying, both in terms of the main and minor components. This fact will condition the reactions that occur in these oils during frying, which will be briefly described in the following section.

## 3. Chemical Reactions Occurring in the Oil During Frying

Frying of foods is considered to be one of the most complex and difficult processes to understand, because of the multitude of reactions taking place and the complexity of the products formed [3]. The high temperatures reached during frying (150–190 °C) along with the exposure of the oil to oxygen, ambient humidity and water released from the food, lead to various chemical reactions to occur in the oil that affect oil’s major and minor components (sterols, tocopherols, squalene, carotenoids, etc.), provoking their degradation and an increase in viscosity, darkening of oil color, formation of foam, etc. In addition, food components, such as trace metals or partial glycerides, among others, can dissolve into the oil, further accelerating its deterioration [59,60]. In shallow frying, only a portion of the food is submerged in oil, and the excess of oil is often discarded after each frying session. In contrast, deep frying involves fully immersing the food in hot oil, which results in a more homogenous heating of the food surface. 

**Figure 1:**
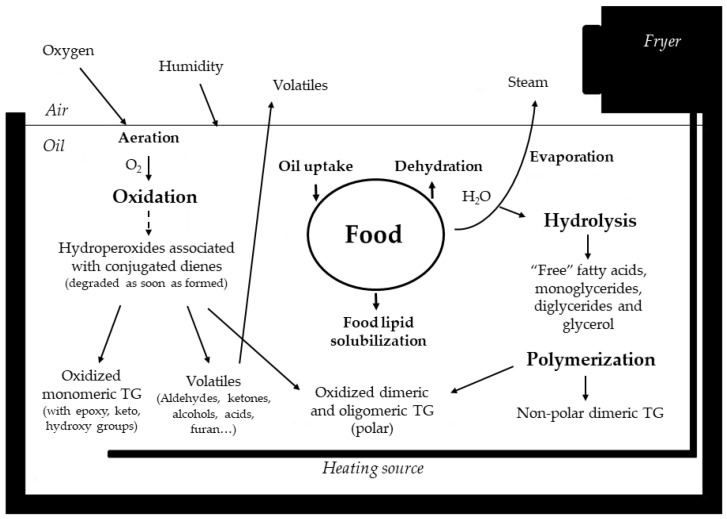
Main physical and chemical phenomena occurring during food deep frying. Adapted from [61,62].

Figure 1 illustrates a simplified overview of the above-mentioned main physical and chemical phenomena occurring in the oil throughout the frying process [61,62]. As can be observed, the main physical phenomena taking place comprise food dehydration, food lipid solubilization and frying oil uptake. Regarding chemical reactions, the main ones are thermoxidation, polymerization and hydrolysis. If temperatures above 200 °C are reached, others like isomerization and cyclization can also occur. All these reactions will be explained in detail in the following Section 3.1, Section 3.2, Section 3.3 and Section 3.4. As a result, a vast variety of compounds of different stability, polarity and molecular weight are generated in the frying oil, with some being volatile and others non-volatile [3,59,63]. The generated compounds are usually classified depending on their polarity [64]. Polar compounds are oxidized TG monomers, dimers and oligomers, volatile compounds, oxidized TG decomposition products and hydrolytic products like fatty acids, monoglycerides (MG) and dyglycerides (DG). Non-polar compounds are cyclic or *trans* isomers of TG monomers, and non-polar TG dimers. Moreover, volatile compounds are very important because they are responsible for the flavor quality of frying oil and fried food, but from a quantitative point of view, they are just a small part of the total number of oil degradation products [3]. The main products generated are non-volatile polar TG dimers and oligomers, followed by oxidized monomeric TG [59].

If oil degradation compounds are generated in significant proportions and absorbed by the fried food, they can negatively affect food sensory properties and shelf life, and most importantly, its safety. As in deep-frying processes at restaurant and industrial level oils are often reused due to economic reasons, the control of heated oil stability and quality is of paramount importance. To avoid the use or reuse of poor-quality frying oils, in most countries, health authorities have established legal limits beyond which the oil must be discarded. The most common limits are based on a maximum percentage of Total Polar Compounds (TPCs) (24–30%) or polymers (16%) [1,65]. The official method for measuring TPCs employs silica gel column chromatography, but as it is time-consuming, other techniques such as High-Performance Size-Exclusion Chromatography (HPSEC) or Fourier Transform Near-Infrared Spectroscopy (FT-NIR) have also been used [65,66,67,68]. In restaurant and industrial settings, easier and faster tests are often used, such as those measuring oil dielectric constant or other test kits, which show certain limitations as previously reviewed [69,70,71]. It is important to note that before reaching the established legal maximum value of TPCs, it has been shown that PUFA-rich oils may contain potentially toxic compounds [3,72].

Moreover, in scientific research studies, different parameters are usually measured or different analytical techniques are employed to evaluate the changes occurring in the physical, chemical and nutritional properties of the frying oils [3,64,65,73,74,75]. Among the physical parameters usually monitored, there are viscosity (as an indirect measure of oil polymerization) and oil color. As for chemical ones, the following can be cited: Iodine Value (IV) or FA composition to study the decrease in the oil unsaturation degree, Acid Value (AV) and “free” fatty acids (FFA%) to study the extent of hydrolysis and *p*-Anisidine Value (AnV) for secondary oxidation compounds. It must be noted that in several studies, the Peroxide Value (PV) and Conjugated Dienes absorption at 231 nm (CD) are also determined as markers of the formation of primary oxidation products. However, as will be explained later, these are unstable compounds that at frying temperatures are degraded as soon as formed, and thus do not accumulate [3]. Primary oxidation compounds can be detected in frying oils during intermittent frying, after their accumulation between frying cycles because the oil temperature decreases. Anyway, all these determinations are performed using classical methodologies, which require large amounts of solvents and offer limited information on the specific nature of the degradation compounds formed. Additionally, more specific analytical techniques can also be employed, such as chromatographic techniques to address the formation of oxidized monomeric, dimeric and oligomeric TG and of volatile compounds, or spectroscopic techniques like Proton Nuclear Magnetic Resonance spectroscopy (^1^H NMR) to study different oxidation compounds. Further research is needed to find better parameters or analytical techniques able to reflect the quality of the oils used for frying, as it will condition the quality of the fried food [65].

Table 2 provides a summary of some studies conducted on oils of different natures submitted to frying conditions in the absence or in the presence of food, using classical methods or more advanced analytical techniques. Those studies were carried out to shed light on the main reactions occurring in oils during frying, which will be briefly described below.

### 3.1. Thermoxidation

**Figure 2:**
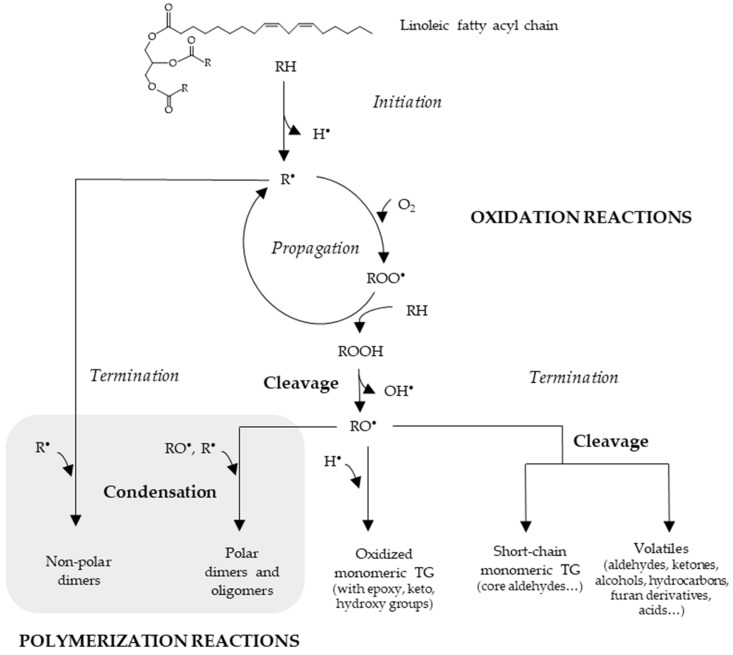
Schematic representation of the main reactions occurring in unsaturated lipids during frying process. Adapted from [59].

Lipid oxidation at high temperatures, which is thermoxidation, is a very complex sequence of oxidative and thermal reactions that occur simultaneously [59,88]. Despite the low availability of oxygen, UFA supported on TG (RH) oxidize, initially forming alkyl free radicals (R*). These reactive species then propagate through a free-radical chain mechanism, which can be classically described in three stages: initiation, propagation and termination [3], as can be observed in Figure 2. It must be noted that at frying temperatures, the initiation stage becomes particularly important because of the low oxygen pressure, which leads to a significant increase in alkyl radicals (R*) with respect to peroxyl radicals (ROO*) [59]. These reactions lead to the formation of hydroperoxides generally associated with conjugated double bonds (ROOH), also called primary oxidation compounds. At temperatures above 100 °C, these latter are present only transiently, indicating that their decomposition rate is higher than that of their formation. This decomposition of ROOH corresponds to the termination stage and evolves through very assorted pathways, alkoxy (RO*) and hydroxyl radicals (*OH) being the main radicals formed. These latter, together with the above-mentioned alkyl radicals (R*), will be involved in several reactions resulting, at the end of the termination stage, in secondary (or further) oxidation compounds, which can be either volatile or non-volatile [3]. Their specific nature is influenced by the FA composition of the oil, linolenic, linoleic and oleic acyl groups being the most significant contributors [89]. Therefore, the choice of frying oil is crucial, as it directly affects food flavor and safety by influencing the production of non-volatile and volatile compounds.

**Figure 3:**
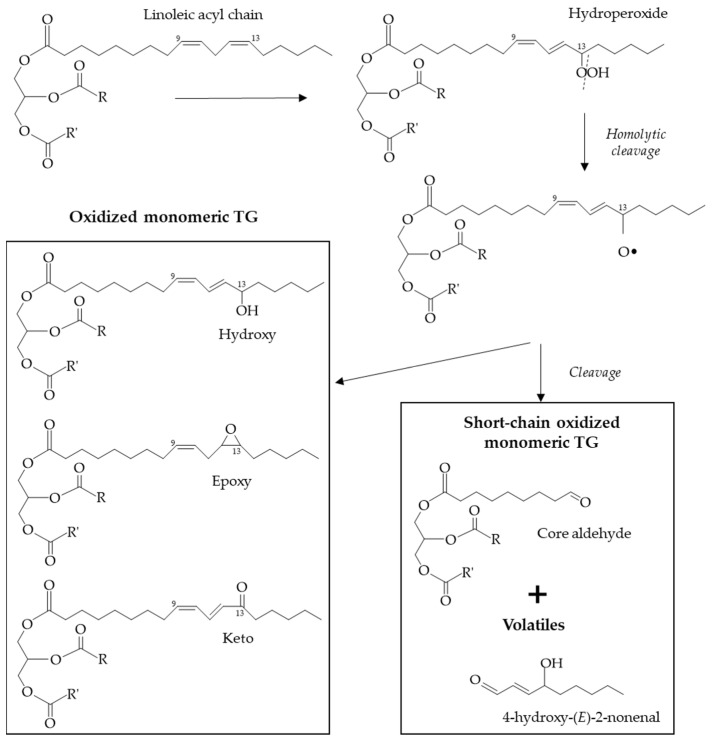
Possible chemical structures that can be generated during frying in the oxidation of linoleic chain (C18:2ω6) supported in position *sn*-1 of triglyceride (TG). R and R’ substituents can be different FA. Adapted from [90].

As illustrated in Figure 2, due to condensation reactions at the termination stage, different polymeric compounds (dimers and oligomers) can be formed in significant amounts, as will be commented on in the next Section 3.2. In addition, at this last stage, oxidized monomeric TG will also be formed, which are characterized by the addition of at least one extra oxygen atom to one or more of the UFA chains, leading to the formation of other oxygenated functional groups. As can be observed in Figure 3, the main ones are epoxy, keto and hydroxy groups, although short-chain glycerol-bound n-oxo aldehydes can also be formed (also known as “core aldehydes”) [59,90]. It has been reported that in heated frying oils that have reached the legal limit of 25% TPC, TG dimers and oligomers account for 12–15% whereas oxidized monomeric TG account for 7–10%, hydrolitic products being of little quantitative significance [59]. As shown in Table 2, they have been extensively studied mainly using chromatographic techniques [67,68,85,91] and have been the subject of several reviews [74,92,93]. Other authors also reported the study of some of these compounds in frying oils by ^1^H NMR spectroscopy, in which no previous chemical modification of the sample is needed [4,78,79,80].

Volatile compounds generated in frying oils are low-molecular-weight compounds. They are mostly removed from oil by steam during deep frying, but the remaining ones are of concern because they can be absorbed by the fried foods, contribute to their flavor and can affect human health. Most abundant volatile compounds generated in oils during frying are aldehydes, although ketones, alcohols, hydrocarbons, acids, lactones and furans are also formed [94]. Extensive research has been conducted on the volatile compounds generated in different oils submitted to frying conditions, to study how oil composition influences the nature and proportions of the volatile compounds generated in the oil, mainly using high-sensitivity analytical techniques, such as Headspace Solid-Phase Microextraction coupled with Gas Chromatography–Mass Spectrometry (HS-SPME-GC/MS) [68,72,81,83].

Although the concentration of volatile compounds is minimal (at parts per million levels), they significantly impact the aroma profile [63]. The nature and concentration of the volatile aldehydes formed, key contributors to aroma, greatly depend on the oil’s FA composition [72,94]. Thus, oils rich in linoleic acyl groups, such as sunflower or soybean oils, tend to mainly generate unsaturated aldehydes like 2,4-decadienal, 2-heptenal, 2-octenal and the saturated aldehyde hexanal. It must be noted that although high levels of linoleic can lead to faster oxidation and potentially undesirable flavors, a minimum proportion of these volatiles is needed to ensure that the distinctive pleasant fried aroma is present in fried food [3,95]. Oils rich in oleic acyl groups, such as EVOO and high-oleic sunflower oil, mainly generate the saturated aldehyde nonanal, and unsaturated ones like 2-decenal and 2-undecenal [68]. Oils rich in linolenic acyl groups, such as linseed, soybean and rapeseed oils, pose specific challenges during frying, as mentioned before, because at high temperatures, they can generate volatile compounds that contribute to undesirable odors, including a “fishy” smell. To mitigate this issue, various oil varieties with reduced linolenic acid content have been developed [38]. In linseed oil submitted to frying conditions, the main aldehydes detected were unsaturated ones, specially 2,4-heptadienal followed by 2-butenal and 2-propenal (acrolein) [72]. It must be noted that in addition to their contribution to aroma, certain volatile compounds generated in frying oils can raise health concerns, such as oxygenated α,β-unsaturated aldehydes like 4-hydroxy-2-nonenal. In this context, regarding the volatile compounds that can be generated during frying, it should be noted that the IARC classified emissions from high-temperature frying as “probably carcinogenic to humans (Group 2A)” [96].

### 3.2. Polymerization

**Figure 4:**
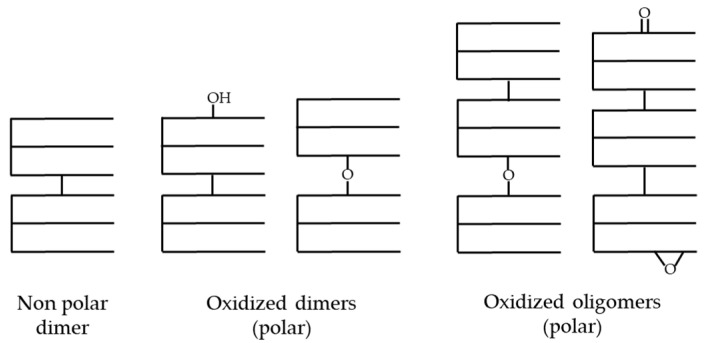
Schematic representation of the molecular structures of polymeric compounds that can be generated during frying. Adapted from [59].

As the temperature rises, the solubility of oxygen decreases, accelerating thermodegradation reactions and promoting the formation of polymeric compounds through interactions between alkyl (R*) and alkoxyl (RO*) radicals in unsaturated acyl groups supported on TG, as can be observed in Figure 2 [3,97,98]. Consequently, TG dimers and oligomers of very different structures are formed, which constitute one of the main and most complex groups of degradation products in frying oils. They can be polar or non-polar, depending on the presence or absence, respectively, of one or more oxygenated functional groups either in the FA chain (e.g., hydroxy, keto, epoxy) or in the linkage between TG (ether or carbon–carbon bond). It must be noted that the presence of non-polar oligomers has not been reported in used frying oils [59]. Figure 4 shows some possible schematic structures of these high-molecular-weight polymeric compounds [59,99].

The formation of TG dimers and oligomers depends on the oil composition, in such a way that higher proportions are generated in oils rich in PUFA than in those rich in MUFA [100]. In addition, their formation is higher, the higher the frying temperature and number of frying cycles [101]. Their generation provokes an increase in oil viscosity, darker oil color and favors oil uptake by food, among others [2,63,102].

To analyze these polymeric compounds, chromatographic techniques are commonly used, and specially HPSEC [67,68,85]. Additionally, oil viscosity is a physical parameter that correlates with the presence of polymers and thus is often monitored due to the simplicity of the measurement [103].

### 3.3. Hydrolysis

In contrast to the above-described degradation reactions, hydrolysis is less complex. When food comes into contact with hot oil, the water content in the food rapidly reaches its boiling point (100 °C), generating steam that interacts with the oil. Consequently, the ester bonds of TG are hydrolyzed, releasing DG, MG, glycerol and “free” fatty acids [63]. The hydrolysis reaction is favored by several factors, including the presence of water and a higher surface-to-volume ratio in the food, high frying temperatures and residual solid particles present in the oil from previously fried foods [104]. It must be noted that this is the same type of reaction that occurs in the digestive tract during the absorption of dietary lipids, but in that case catalyzed by digestive enzymes. Although the amounts of hydrolytic products released may not be significant in terms of quantity, they play a crucial role in the degradation of frying oils and thus affect the quality of fried foods. These fatty acids, MG and DG oxidize more readily than when they are still part of the original TG structure, accelerating the deterioration of the oil and decreasing the oil’s smoke point, which in turn promotes smoke formation [3,7].

The extent of hydrolysis is commonly assessed by measuring the FFA% and Acid Value (AV) of frying oils, as shown in Table 2 [85,86,87]. Moreover, hydrolytic compounds in frying oils can also be quantified using chromatographic techniques like HPLC [64] and spectroscopic ones like ^1^H NMR [4,79,80].

### 3.4. Other Reactions

During frying, in addition to the above-described reactions, others like isomerization and cyclization can also occur in UFA chains [59]. **Isomerization** reactions provoke the formation of *trans* FA, which are UFA with at least one double bond in the *trans* configuration. It must be noted that in nature, most of the double bonds of UFA are in the *cis* configuration. Although *trans* FA can occur naturally in meat and milk from ruminant animals in small proportions, they are mostly generated during oil processing by partial hydrogenation or by severe thermal treatments (> 200 °C) [105], such as the deodorization step during the refining of vegetable oils and frying processes. It must be noted that the consumption of *trans* FA generated during oil processing has been associated with detrimental health effects [22,47,106].

The formation of *trans* FA during deep frying tends to increase with higher frying temperatures and longer cooking times [67,84], so shorter frying times and lower temperatures are highly recommended. Anyway, isomerization reactions during frying have been scarcely studied, in comparison with the reactions described above. This is probably due to the fact that their main source in fried food is the use of hydrogenated oils for frying, and not their formation as a consequence of frying conditions [59].

On the other hand, **cyclization** reactions can also occur in UFA during frying at temperatures above 200 °C, but to a low extent [22,107,108]. The nature of the cyclic compounds formed depends on the precursor FA, in such a way that it has been reported that linolenic gives rise to unsaturated rings (cyclopentenyl and cyclohexenyl), linoleic to cyclopentyl, cyclopentenyl, cyclohexyl and bicyclic rings, and oleic to saturated rings (cyclopentyl and cyclohexyl) [107]. These non-polar compounds are usually studied by chromatographic techniques and are considered as potentially toxic [64,108]. In addition to cyclic FA, this type of reaction could also lead to the generation of polycyclic aromatic hydrocarbons (PAHs) [109].

Taking into account the complexity of the reactions previously described and of the products generated in them, it is not surprising that the food frying process is considered one of the most complex in the field of food chemistry [3].

## 4. Factors Conditioning Frying Medium Degradation

The above-described changes occurring in the oils during frying are affected by several factors, such as the oil’s nature (major and minor components), the frying conditions employed (temperature, time…) and the characteristics of the food immersed in the hot oil [63,110]. These factors are briefly described below.

### 4.1. Oil Composition

#### 4.1.1. Unsaturation Degree of FA and Its Influence on Oil Oxidative Stability

Regarding the oil composition, the factor that most influences degradation reactions during frying is the degree of unsaturation of the main FA present in the oil. As previously commented, the more polyunsaturated an oil composition is, the more prone it is to undergo thermoxidation and polymerization reactions [3]. Therefore, among the oils available in the market, those rich in MUFA and with a low content in PUFA, especially linolenic FA (<3%), are highly recommended for frying, such as olive oil (see Table 1) [3,7]. The abundance of monounsaturated oleic FA gives to these MUFA-rich oils greater resistance to degradation, in comparison with other oils rich in PUFA, such as sunflower oil, whose primary FA is linoleic acid, or soybean oil, rich in linoleic and linolenic acid [111,112,113]. For example, in some studies in which EVOO (~82% oleic), sunflower oil (~55% linoleic) and linseed oil (~50% linolenic) were subjected to frying conditions in the absence of food, it was observed that the legal limit of 25% Total Polar Compounds (TPCs) was reached after 33 h in olive oil, 17 h in sunflower oil (it degraded twice as fast as olive oil) and nearly 4 h in linseed oil (it degraded almost 10 times faster than olive oil] [78,79,80]. Furthermore, the volatile aldehydes generated in the three oils were studied after 20 h of heating and it was evidenced that a lower number and abundances were formed in olive oil, in comparison with sunflower and linseed oils. It is worth noting that the potentially toxic 4-hydroxy-2-nonenal and 4-hydroxy-2-hexenal, derived from linoleic and linolenic chains, respectively, [114] were not detected in olive oil, but they were generated in the other oils [72].

As indicated before, nowadays, in addition to vegetable oils naturally rich in MUFA, industry provides other options, like high-oleic modified oils (high-oleic sunflower, high-oleic soybean or high-oleic corn oils, among others) [36].

#### 4.1.2. Length of FA and Content of “Free” Fatty Acids: Influence on Oil Smoke Point

The smoke point is the temperature at which oil begins to smoke. Its appearance indicates that TG are hydrolyzing into “free” fatty acids and glycerol, and that glycerol (1,2,3-propanetriol) leads to the formation of acrolein (2-propenal) after water elimination [115,116]. Acrolein is one of the main components of the bluish smoke, which is capable of irritating the skin and mucous membranes. The influence of the unsaturation degree of the oil on the smoke point is minimal, although it is known that acrolein is also formed in PUFA oxidation [115]. The smoke point depends mainly on the presence of “free” fatty acids and also on the molecular weight of FA, in such a way that oils containing short-chain FA, like lauric acid (C12)-rich oils (coconut or palm kernel oils), have a lower smoke point than those rich in longer-chain FA of C16 or C18 (see Table 1) [116].

Throughout the frying process, the smoke point of the oil decreases due to the release of more and more FA and glycerol, as TG hydrolysis progresses in the presence of water from food, and also to the presence of thermodegradation compounds [116]. Decades ago, the smoke point of each type of oil was also taken into account for the evaluation of its suitability for frying. However, nowadays, it is considered that the smoke point is not a precise indicator of oil susceptibility to degradation, nor of the degradation process that the oil is undergoing [117].

#### 4.1.3. Oil Minor Components and Their Influence on Oil Oxidative Stability

The stability of oil during frying depends not only on its major components (especially the degree of unsaturation of the FA supported on TG, as described above), but also on the presence of other minor components naturally present (tocopherols, sterols, polyphenols, …) or added as antioxidant additives, which could exert an antioxidant effect and improve the oil’s frying performance. Moreover, it is worth noting that these minor compounds can also be absorbed by the fried food and thus ingested [23]. Special attention was paid to this topic in foods fried in EVOO, as its polyphenols are associated with positive effects on cardiovascular health among others [10,24,25,118]. Several reviews were focused on the evolution during frying of these antioxidant oil minor components, either naturally present or added [119,120,121]. It must be noted that their effect depends on their chemical structure and amount present in the oil. In the last few decades, the growing concern of consumers about the possible toxicity of antioxidant additives of synthetic origin and the increasing demand for food containing bioactive compounds have led the food industry to focus on alternative additives of natural origin, mainly coming from vegetable sources [122].

On the contrary, it must be noted that other oil minor components can negatively affect oil’s frying stability if present in enough concentrations [7]. Trace metals like iron and copper can exert a prooxidant activity. Phospholipids and partial glycerides (MG, DG) can cause excessive foaming [119], and this foam could be stabilized by TG oxidation products. As stated before, in order to reduce their content, most types of vegetable oils used for frying are submitted to a refining process before commercialization.

### 4.2. Frying Conditions

As frying conditions greatly affect oil and fried food quality, it is necessary to pay attention to their influence and to control the parameters during the whole process [123]. Regarding the oil temperature and frying time, it is well known that higher temperatures and prolonged times significantly accelerate the degradation of oil [7,110,124]. In this sense, the optimum frying temperature is considered to be 150–190 °C, and the optimum time will depend on the food characteristics. Reusing oil in intermittent or discontinuous frying implies that the oil is subjected to repeated cooling and heating cycles, which contributes to oil degradation (formation of the compounds described in the previous section, color darkening, increased viscosity and foaming) [125]. In addition, other factors should be taken into account, such as the food–oil ratio, oil filtering and/or skimming regularly to remove food debris and oil exposure to oxygen, which can be minimized by covering the oil tank with a lid between cycles but favored in case of excessive fume extraction [3,7,124].

### 4.3. Food

The characteristics of the food immersed in the hot oil greatly affect the complex chemical and physical reactions occurring during frying: food composition (content of water, proteins, carbohydrates, lipids or certain minor components), food size (dimension) and surface properties, the application of coatings or pre-treatments to food (blanching, air drying…), among others. The influence of food itself on the changes occurring in the frying medium is very different depending on the intrinsic characteristic of foods [126,127,128,129].

Thus, it is evident that the oil degradation level reached during frying is greatly affected by the aforementioned factors. Therefore, it is essential to control them to guarantee obtaining high-quality, healthy and safe fried foods.

## 5. Fried Food Quality and Health Implications

There is a wide variety of foods that can undergo the frying process: vegetables, meat, fish or fruits, either in raw state or after undergoing batter coating, breading or previous treatments such as cutting, drying, etc. [129,130]. Table 3 summarizes the main changes that food components can undergo during frying, contributing to the unique sensory qualities of fried foods: golden and crusty exterior, juicy interior and appealing aroma and taste [131,132,133].

In summary, water evaporates, creating pores and crispness in the surface; starch gelatinizes inside; and proteins denature and participate in Maillard-type reactions with reducing sugars or lipid oxidation carbonyls, enhancing flavor and color. Additionally, lipids can also interact in Maillard-type reactions by the amino group of the polar head of some phospholipids, like phosphatidylethanolamine and phosphatidylserine. There is a lipid exchange between food and the frying medium, because food can absorb oil, increasing its caloric content, and simultaneously food lipids can solubilize in the frying medium. As mentioned before, if reused oils rich in PUFA are employed, food also absorbs the potentially toxic compounds present in the oil. Food mineral content is scarcely affected during frying. Regarding food vitamins, as the temperature inside never exceeds 100 °C, as long as there is some liquid water left in it and the frying time is short, heat-sensitive vitamins are expected to be moderately reduced (like vitamin C or thiamine). As for food lipid-soluble vitamins, such as vitamin E (tocopherols) or provitamin A carotenoids, they can leach into the frying medium or be oxidized. At this point, it must be noted that if vegetables oils rich in vitamin E (tocopherols) (see Table 1) or rich in bioactive polyphenols like EVOO are employed, due to oil uptake phenomena, fried food is enriched in these components, which is beneficial from a nutritional point of view [131,132,134].

### 5.1. Fried Food Lipids: A Result of Oil Uptake and Food Lipid Solubilization

Several reviews have been devoted to the study of oil absorption by food during frying, and specially on how to reduce it [21,102,124,135,136,137]. This interest is due to its implications in the nutritional quality and safety of fried food, and consequently, on human health. It has been estimated that oil absorption in fried potatoes can reach up to 40% by weight [59].

The previously mentioned water loss that food experiences during frying leads to the formation of pores, cavities and channels within the food structure, allowing the frying oil to penetrate. This absorption mainly occurs during the cooling of the fried food, and to a lesser extent during frying itself, as the pressure gradient created by the evaporation of water inside the food limits oil penetration to larger cavities [110,130,131]. Furthermore, as the process continues, the crust formed on the surface thickens and impedes oil movement into the food [138,139]. However, once the food is removed from the fryer or pan and while it cools, internal pressure decreases due to the condensation of steam within the food, creating a sort of “vacuum” that facilitates the absorption of oil that was adhered to the surface. Studies indicate that even with prolonged frying times, there is no diffusion of oil to the center of the food [110]. Oil is estimated to penetrate only about 1 mm deep into fried food [140]. Additionally, it is worth noting that quickly drying the surface of freshly fried food can significantly reduce the final lipid content [139]; otherwise, during cooling, the amount of oil adhered to the surface may occupy the pores and channels created in the crust.

Oil absorption in food depends on various factors, including the oil quality, frying temperature and time, as well as the type of food and its compositional characteristics, shape and porosity [110,141]. In this regard, studies have shown that the absorption of oil is greater when frying in degraded oils [142]. Oil adhesion to the food surface is higher in degraded oils, not only due to their greater viscosity, but also because the presence of degradation products with higher polarity reduces the surface tension between the oil and the food. Additionally, some authors have indicated that the oil adhered to the surface of fried food may be more oxidized than the frying medium itself, as the more polar oxidation products facilitate adhesion [139]. If the frying temperature is below the optimal value for a specific type of food, greater oil absorption occurs, and the fried food may become greasy [21,143]. This is because lower temperatures result in less desirable vapor pressure generated inside the food, favoring absorption. Reducing the thickness of the food to be fried increases the surface-to-volume ratio, which promotes oil adsorption on the food surface [144,145]. Water loss from the food during frying is also related to oil absorption, in such a way that a higher initial water content leads to greater evaporation and pore formation, resulting in increased oil uptake [143,145,146]. Foods with a low lipid content (e.g., potatoes) tend to absorb more oil compared to foods rich in lipids (e.g., salmon), which may even see a decrease in the lipid content after frying due to the melting of their fats and migration into the frying medium [147]. Greater oil absorption has also been observed when the coating mixtures used (batter, breading or flouring) contain chemical leavening agents (sodium bicarbonate) that promote gas formation, consequently enhancing surface porosity [148]. Conversely, if these coating mixtures contain water-retaining ingredients (such as cellulose or gums), oil uptake will be reduced. Other processes prior to frying, such as pre-frying or drying the food, can significantly reduce oil absorption [126,137].

### 5.2. Sensory Properties of Fried Food

Frying provides food with a crispy dehydrated surface texture and a juicy interior, along with a unique aroma and golden color that other cooking methods cannot achieve [149]. Fresh or minimally reused oils yield the best sensory characteristics [130].

The texture of fried food can be influenced by different factors, such as the type of frying oil, oil temperature and the characteristics of the fried product. A correlation between the oil’s FA profile and fried food texture has been reported, noting that the hardness increases with higher SFA levels in the oil; on the contrary, higher frying temperatures reduce the hardness [150,151].

The flavor of fried foods is developed through a combination of reactions between compounds initially present in food and those absorbed from the frying oil. During the development of Maillard-type reactions, sugar caramelization and frying oil oxidation, hundreds of compounds of a different nature are generated, including brown pigments (melanoidins), volatiles and savory compounds responsible for fried flavor [132,152,153]. The high temperatures of the process and water evaporation on the food surface favor these types of reactions, involving food proteins and reducing sugars present on the surface, as well as certain lipid oxidation products generated in the frying medium. It is estimated that between 30 and 60% of the volatile compounds responsible for the aroma of fried foods derive from oil degradation [126]. Nevertheless, the food itself also contributes to the final aroma by containing important precursors of aromatic substances, including sulfur-containing amino acids and thiamine [126]; that is why sometimes it is possible to identify solely by smell the nature of the food being fried. Key factors influencing fried food flavor include the type of oil, storage conditions, frying temperature and time and food characteristics (moisture content, size, surface, pre-frying treatments), among others [132]. The main volatile compounds generated from the main FA present in vegetable oils (linolenic, linoleic and oleic) were indicated before in Section 3 [154].

As for the characteristic golden color of fried foods, this is the result of the different chemical reactions that take place in the food, mainly non-enzymatic browning reactions, such as Maillard-type ones and the caramelization of sugars [134]. The high temperatures of the process and the evaporation of water on the surface of the food favor this type of reaction, in which food proteins and reducing sugars present on the surface are involved, as well as certain lipid oxidation products generated in the frying medium itself. Melanoidins are the main ones responsible for the darkening of fried food. It must be noted that in the last few years, especial attention was paid to excessive browning in carbohydrate-rich foods, like potatoes, because of its relation with the presence of acrylamide, a potentially toxic Maillard reaction product [155]. The extent of fried food color change depends on several factors, in such a way that a higher frying temperature and time [156] and higher frying oil degradation levels [84,157] have been associated with greater color changes in fried food. In addition, substrates of the above-mentioned reactions will depend on food composition.

### 5.3. Health Implications

Regarding the potential relationship between the consumption of fried foods and the incidence of prevalent diseases, like cancer or cardiovascular diseases, several epidemiological studies have been carried out, although variable results were obtained [158,159]. It must be noted that in many of these studies, the composition of the frying oil was not studied in depth and the associations reported were attributed to compounds that were not derived from the oil degradation itself, but from other reactions, such as acrylamide formed in carbohydrate-rich foods or heterocyclic amines in meat.

In this sense, many other studies focused on used frying oils and their nutritional and physiological effects, which is a very challenging task due to different reasons [160]. Firstly, a wide variety of oil degradation products can be generated depending on the oil composition and frying conditions and the proper identification of these compounds is not always carried out. Secondly, in many studies, extremely thermally abused oils were administered to animal models, which is far away from the real conditions of normal culinary practices. And thirdly, the distinction between the oxidized compounds coming from the diet and those formed in vivo is not always easy to achieve.

In the last few years, it has been highlighted that when PUFA-rich oils are used for frying, several lipid oxidation products of a different nature are generated, such as epoxy-FA and aldehydes, which may pose substantial health hazards [161]. Special attention was paid to oxidized TG monomers due to their possible detrimental health effects given their high absorbability [160]. These oxidized monomeric TG remain in the oil and can be absorbed by the food, and thus be ingested [3,59,97]. In addition, there is increasing concern about oxygenated α,β-unsaturated aldehydes, like linoleic-derived 4-hydroxy-2-nonenal and also linolenic-derived 4-hydroxy-2-hexenal, which can also be absorbed by food and have been related to several diseases, like cancer and Alzheimer’s, among others [114,162]. When EVOO, sunflower and linseed oils were submitted to frying conditions for 20 h, 4-hydroxy-2-nonenal was detected in sunflower oil and 4-hydroxy-2-hexenal in linseed oil, but none of them in EVOO [72], which is in total agreement with the composition of the oils in the main acyl groups.

Moreover, most of these aldehydes generated in PUFA-rich oils are volatile and can be present, among other compounds, in frying oil fumes, which in turn have been linked to decreased lung function, significantly increasing the risk of lung cancer [163,164]. In light of these findings, the IARC classified high-temperature frying emissions as “probably carcinogenic to humans” (Group 2A) [96]. High frying temperatures, the repeated use of oils rich in PUFA and inadequate ventilation lead to the release and accumulation of harmful volatile compounds. In addition to aldehydes, these fumes can contain benzene, polycyclic aromatic hydrocarbons (PAHs) and heterocyclic amines, all of which are associated with various health risks [123].

The need to study the fate of frying oil degradation compounds that are not absorbed in the gastrointestinal tract, like TG dimers and oligomers, which could affect gut mucosa and microbiota, has also been pointed out [158].

## 6. Conclusions

In summary, this review presented a comprehensive overview of the composition in the main and minor components of the vegetable oils most commonly used for frying worldwide. This composition greatly affects the complex chemical reactions occurring in the oils during frying, predominantly thermoxidation and polymerization, with hydrolysis being less significant. Additionally, if temperatures above 200 °C are applied, isomerization reactions resulting in the formation of *trans* fatty acyl chains, as well as cyclization reactions, can also occur. The extent of all the above-mentioned reactions depends on several factors, which were also summarized, and must be controlled to obtain high-quality and safe fried foods. Moreover, the potential health implications associated with the consumption of fried food were briefly addressed. Despite the above-mentioned health concerns, the moderate consumption of fried foods can be safely included in a balanced diet, always following good frying practices. The lipid composition of the food changes during frying and often resembles that of the frying medium used; therefore, the choice of the frying oil is crucial. As mentioned before, high-quality oils, such as EVOO, are particularly recommended for their stability and health benefits due to their high content in MUFA (oleic) and in minor antioxidant components, like tyrosol and hydroxytyrosol. Implementing good frying practices, such as maintaining a temperature below 180 °C, pre-drying foods and minimizing oil reuse, can reduce harmful compound formation.

As heated PUFA-rich oils may contain potentially toxic compounds before reaching the established legal maximum value of TPCs, further research is needed to find better parameters or analytical techniques able to monitor and reflect the quality of the oils used for frying, as it will condition the quality of the fried food. In addition, in the last few decades, special efforts have been made to find effective strategies to enhance frying oil stability, such as the addition of compounds or extracts with potential antioxidant activity, especially those of natural origin. It should be noted that this is a challenging task, because due to the high temperatures applied, the degradation of the added antioxidants can also occur, thus reducing the expected effect.

## Figures and Tables

**Table 1:** Composition of several vegetable oils in main fatty acyl chains (FA), expressed as percentage of total FA, together with that of some minor components, expressed as mg/kg crude oil [14,15].

Vegetable Oils		Olive	High-Oleic Sunflower	Rapeseed *	Peanut	Rice Bran	Sunflower	Maize (Corn)	Soybean	Cottonseed	Palm	Palm Kernel	Coconut
**Fatty acyl chains (FA)**													
**Monounsaturated fatty acyl chains (MUFA)**													
Palmitoleic	16:1ω7	0.3–3.5	nd–0.1	nd–0.6	nd–0.2	nd–0.5	nd–0.3	nd–0.5	nd–0.2	nd–1.2	nd–0.6	nd–0.2	nd
Oleic	18:1ω9	55.0–83.0	75.0–90.7	51.0–70.0	35.0–80.0	38.0–48.0	14.0–43.0	20.0–42.2	17.0–30.0	14.7–21.7	36.0–44.0	12.0–19.0	5.0–10.0
Eicosenoic	20:1ω9	nd–0.4	0.1–0.5	0.1–4.3	0.7–3.2	nd–0.8	nd–0.3	0.2–0.6	nd–0.5	nd–0.1	nd–0.4	nd–0.2	nd–0.2
Erucic	22:1ω9	nd	nd–0.3	nd–2.0	nd–0.6	nd	nd–0.3	nd–0.3	nd–0.3	nd–0.3	nd	nd	nd
**Polyunsaturated fatty acyl chains (PUFA)**													
Linoleic	18:2ω6	3.5–21.0	2.1–17.0	15.0–30.0	4.0–43.0	21.0–42.0	45.4–74.0	34.0–65.6	48.0–59.0	46.7–58.2	9.0–12.0	1.0–3.5	1.0–2.5
Linolenic	18:3ω3	nd–1.5	nd–0.3	5.0–14.0	nd–0.5	0.1–2.9	nd–0.3	nd–2.0	4.5–11.0	nd–0.4	nd–0.5	nd–0.2	nd–0.2
**Saturated fatty acyl chains (SFA)**													
Caprylic	8:0	nd	nd	nd	nd	nd	nd	nd	nd	nd	nd	2.4–6.2	4.6–10.0
Capric	10:0	nd	nd	nd	nd	nd	nd	nd	nd	nd	nd	2.6–5.0	5.0–8.0
Lauric	12:0	nd	nd	nd	nd–0.1	nd–0.2	nd–0.1	nd–0.3	nd–0.1	nd–0.2	nd–0.5	45.0–55.0	45.1–53.2
Myristic	14:0	nd–0.1	nd–0.1	nd–0.2	nd–0.1	nd–1.0	nd–0.2	nd–0.3	nd–0.2	0.6–1.0	0.5–2.0	14.0–18.0	16.8–21.0
Palmitic	16:0	7.5–20.0	2.6–5.0	2.5–7.0	5.0–14.0	14.0–23.0	5.0–7.6	8.6–16.5	8.0–13.5	21.4–26.4	39.3–47.5	6.5–10.0	7.5–10.2
Stearic	18:0	0.5–5.0	2.9–6.2	0.8–3.0	1.0–4.5	0.9–4.0	2.7–6.5	nd–3.3	2.0–5.4	2.1–3.3	3.5– 6.0	1.0–3.0	2.0–4.0
Arachidic	20:0	nd–0.6	0.2–0.5	0.2–1.2	0.7–2.0	nd–0.9	0.1–0.5	0.3–1.0	0.1–0.6	0.2–0.5	nd–1.0	nd–0.2	nd–0.2
Behenic	22:0	nd–0.2	0.5–1.6	nd–0.6	1.5–4.5	nd–1.0	0.3–1.5	nd–0.5	nd–0.7	nd–0.6	nd–0.2	nd–0.2	nd
Lignoceric	24:0	nd–0.2	nd–0.5	nd–0.3	0.5–2.5	nd–0.6	nd–0.5	nd–0.5	nd–0.5	nd–0.1	nd	nd	nd
**Minor components (mg/kg)**													
Total sterols		1000–2000	1700–5200	4500–11,300	900–2900	10,500–31,000	2400–5000	7000–22,100	1800–4500	2700–6400	300–700	700–1400	400–1200
Total tocopherols and tocotrienols		55–320	450–1120	430–2680	170–1300	191–2349	440–1520	330–3720	600–3370	380–1200	150–1500	nd–260	nd–50

* Canola low-erucic acid; nd: not detectable (≤0.05%).

**Table 2:** Parameters or compounds commonly studied in several oils using different methodologies and frying conditions, either in the absence or in presence of food.

Oil	Fried Food	Frying Conditions	Parameters or Compounds Studied in Oils	Methodology	Ref.
Cottonseed, sunflower, palm, shortening, virgin olive (2 L)	Potato (400 g)	170 °C, 8–9 min, 8 cycles	Polymerized TG Oxidized FA (epoxystearates, epoxyoleates, ketostearates)	HPSEC FAME and GC/MS	[76]
Refined canola (3.75 L)	Frozen par-fried French fries (200 g)	185 °C, 215 °C, 5 min, 56 cycles	TPC (DG, oxidized TG, dimers and polymers) FA composition, *trans* FA AnV Oil color	Gravimetric method HPSEC FAME and GC/MS UV-Vis UV-Vis	[67]
EVOO, peanut, canola (1.5 L)	French fries (50 g)	175 °C, 6 min, 16 cycles	TPC (dimeric, polymeric and oxidized monomeric TG) Volatiles (aldehydes, hydrocarbons, ketones, alcohols, carboxylic acids, furans) AnV	Dielectric constant, HPSEC HS-SPME-GC/MS UV-Vis	[68]
High-oleic sunflower (3.3 kg)	Potato chips (200 g)	175 °C, 3 min, 40 cycles	Total oxylipin concentrations (FA with hydroperoxy, hydroxy, epoxy, dihydroxy groups)	LC-MS	[77]
EVOO, sunflower, virgin linseed (4 L)	None	190 °C, 8 h/d, 5 days	FA composition, IV and degradation compounds (aldehydes, epoxides, MG, DG) TPC	^1^H NMR Dielectric constant	[78,79,80]
EVOO, soybean, sunflower (4 L)	Doughnuts (40 g), pork adipose tissue (250 g), salmon (250 g)	190 °C, 1 min, 8 h/d for 4 days	FA composition and degradation compounds (aldehydes, epoxides, alcohols, MG, DG)	^1^H NMR	[4]
EVOO, refined sunflower, virgin linseed (4 L)	None	190 °C, 20 h (8 h/d)	Aldehydes (alkanals, alkenals, alkadienals, alkatrienals, oxygenated saturated and α,β-unsaturated aldehydes) TPC	HS-SPME-GC/MS Dielectric constant	[72]
Sunflower, high-oleic sunflower, rapeseed, high-oleic rapeseed, palm olein (1.5 L)	French fries (175 g)	170 °C, 4 min; 36 h, 12 cycles	Volatiles (alkanals, 2-alkenals, 2,4-alkadienals, alcohols, ketones) 4-hydroxy-2-(*E*)-nonenal TPC PV AnV Polymerized TG	HS-SPME-GC DHS-GC/MS DGF C-III 3e DGF C-VI 6a DGF C-VI 6e Gel Permeation Chromatography	[81]
Palm, rapeseed, sunflower, soybean (0.6 L)	French fries (160 g), pork loin strips (160 g)	173–182 °C, 10 min	Volatile aldehydes in cooking oil fumes (alkanals, 2-alkenals, 2,4-alkadienals)	HPLC-UV	[82]
Coconut, soybean, olive, vegetable shortening (4 L)	Potato chips	180 °C, 4 min; 80 cycles	FA composition Tocopherols Free radical scavenging activity Volatiles (alkanals, 2-alkenals, 2,4-alkadienals) Color AV AnV CD (234 nm) TPC (490 nm)	FAME and GC/FID HPLC/FD DPPH method HS-SPME-GC/MS Colorimeter Titration UV-Vis UV-Vis UV-Vis	[83]
Olive pomace, and blended with coconut (2.7 L)	French fries (200 g)	180 °C, 9 min, 60 cycles	FA composition IV Sterols TPC, polymeric TG, oxidized monomeric TG, AnV, AV, color, *trans* FA Oxidative stability	FAME and GC/FID FT-NIR according to AOCS Cd 1e-01 Thin-layer chromatography FT-NIR according to DGF C-VI 21 Rancimat	[84]
Olive pomace, and blended with coconut (2.7 L)	French fries (200 g)	180 °C, 9 min, 60 cycles	FA composition Tocopherols TPC TG dimers, oligomers, oxidized TG monomers, DG, MG, free FA	FAME and GC/FID GC/FID Dielectric constant, HPSEC HPSEC	[85]
Palm, peanut, camellia (2 L)	Potatoes (80 g)	170 °C, 3 min, 75 cycles	FA composition Tocopherols AV IV PV AnV	FAME and GC/FID HPLC-FD Titration Titration Titration UV-Vis	[86]
EVOO, virgin olive, olive, sunflower (1.5 L)	Potato chips (300 g)	170 °C, hourly, 9 h/d	FA composition Tocopherols and tocotrienols *Beta*-carotene Total phenols TPC FFA and PV AnV K_232_ and K_270_ Oxidative Stability	FAME and GC/FID HPLC-FD UV-Vis (454 nm) Folin–Ciocalteu Dielectric constant Titration UV-Vis UV Rancimat	[87]

Abbreviations: AOCS, American Oil Chemists’ Society Official Methods; DGF, Deutsche Gesellschaft für Fettwissenschaft Methods; DHS, Dynamic headspace; DPPH, 2,2-Diphenyl-1-picrylhydrazyl assay; FAME, fatty acid methyl ester; FD, Fluorescence detector; FID, Flame Ionization detector; FT-NIR, Fourier Transform Near-Infrared Spectroscopy; LC, Liquid chromatography; UV–Vis, Ultraviolet–Visible Spectrometry.

**Table 3:** Main changes that can occur in food components during frying. Adapted from [131,132,133].

Component	Main Changes Caused by Frying
**Water**	- Significant loss due to evaporation on the food surface.
**Lipids**	- Changes in total content and composition due to lipid exchange: food typically absorbs oil, and simultaneously food lipids can solubilize into the frying medium. - Oxidation of food lipids. - Participation in non-enzymatic Maillard-type browning reactions, through the carbonyl group of some lipid oxidation products, like aldehydes, and/or the amino group of the polar head of some phospholipids, such as phosphatidylethanolamine and phosphatidylserine.
**Carbohydrates**	- Participation of reducing sugars through their carbonyl group in the Maillard reaction along with the amino group of proteins or the above-mentioned phospholipids. - Starch gelatinization within the interior of the food.
**Proteins**	- Participation in Maillard reactions with reducing sugars and Maillard-like reactions with oil degradation products. - Denaturation.
**Vitamins**	- Moderate losses of heat-sensitive vitamins. - Leaching of fat-soluble vitamins into the frying medium (water soluble vitamins cannot).
**Minerals**	- No significant losses.

## Data Availability

No new data were created or analyzed in this study. Data sharing is not applicable to this article.
